# Cardiac Angiosarcoma With Pulmonary Metastasis: A Rare and Challenging Case

**DOI:** 10.7759/cureus.43962

**Published:** 2023-08-23

**Authors:** Adesola A Agboola, Adetola F Oshikoya, Oluwatobiloba F Fasoranti-Sowemimo, Priyanka Sachdev, Iqra Samreen, Chinyere L Anigbo, Muhammad Haseeb, Hira Nasir

**Affiliations:** 1 Pathology and Laboratory Medicine, Dele Hospitals Lagos, Lagos, NGA; 2 Internal Medicine, Near East University, Nicosia, CYP; 3 General Practice, General Hospital Odan, Lagos, NGA; 4 Medicine and Surgery, Obafemi Awolowo University, Ile-Ife, NGA; 5 Internal Medicine, Liaquat University of Medical and Health Sciences, Jamshoro, PAK; 6 Internal Medicine, Deccan College of Medical Sciences, Hyderabad, IND; 7 Internal Medicine, University of Nigeria, Enugu, NGA; 8 Internal Medicine, Allama Iqbal Medical College, Lahore, PAK; 9 Internal Medicine, Bahria International Hospital Lahore, Lahore, PAK; 10 Internal Medicine, Mayo Hospital, Lahore, PAK

**Keywords:** metastatic cardio angiosarcoma, pulmonary metastasis, primary cardiac angiosarcoma, cardiac tumor in adults, primary cardiac tumor

## Abstract

Cardiac angiosarcoma is a rare and aggressive malignant tumor arising from the endothelial cells of the heart. It accounts for only a small fraction of all cardiac neoplasms and has a poor prognosis. We present a challenging case of a 20-year-old student who presented exertional dyspnea, palpitation, and occasional chest discomfort. Her clinical picture, radiological and pathological investigations confirm the diagnosis of cardiac angiosarcoma with pulmonary metastasis. This case highlights the importance of early diagnosis and multidisciplinary management for improved patient outcomes.

## Introduction

Cardiac angiosarcoma is an extremely rare primary cardiac tumor, accounting for less than 2% of all primary cardiac neoplasms [1]. It originates from the endothelial cells lining the heart and is characterized by rapid growth, early metastasis, and a dismal prognosis. The most common sites of metastasis include the lungs, liver, and brain [2]. Almost 25% of cardiac neoplasms are malignant, which include lymphoma, sarcoma, and mesothelioma. Cardiac angiosarcoma is an uncommon malignant neoplasm and presents significant diagnostic challenges, often leading to delayed diagnosis and poor treatment outcomes [1-3]. Here, we present a case of cardiac angiosarcoma with pulmonary metastasis emphasizing the early diagnostic approach, treatment strategies, and challenges faced during management.

## Case presentation

**Figure 1:**
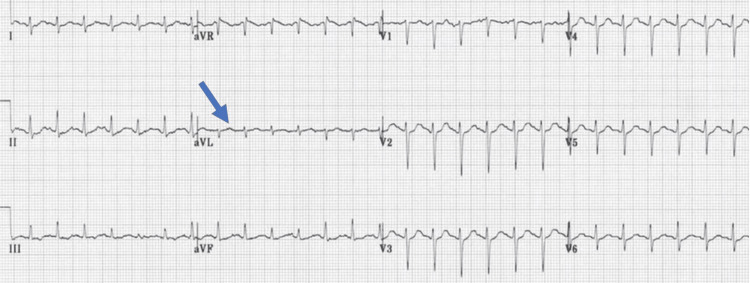
Electrocardiogram demonstrating sinus tachycardia.

A 20-year-old medical student presented with a three-month history of progressive dyspnea on exertion associated with palpitations and occasional chest discomfort. Dyspnea was gradual in onset and progressive, followed by intermittent palpitations and occasional chest discomfort. She denied any significant weight loss, fever, or other constitutional symptoms. She had no history of family disease. She had no history of smoking, alcohol abuse, or illicit drug use. On physical examination, she was anxious, afebrile, and oriented to time, place, and person with mild tachycardia (110/minute), blood pressure of 110/85 mmHg, respiratory rate of 21/minute, and oxygen saturation of 93% on room air. On chest auscultation, there were coarse crepitations in both lung bases, and a systolic murmur of grade II/VI was audible at the cardiac apex. There was no peripheral edema or jugular venous distention, and the rest of the systemic clinical examination was unremarkable. An urgent electrocardiogram (ECG) showed sinus tachycardia and non-specific T-wave abnormalities (Figure 1). The chest x-ray demonstrated a right lower lobe infiltrate with a slight mediastinal shift. Her detailed laboratory evaluations were significant for leukocytosis, elevated erythrocyte sedimentation rate, and C-reactive protein. Arterial blood gas analysis revealed type-I respiratory failure (Table 1). Autoimmune screening and workup for tuberculosis and fungal infections were also negative.

**Table 1:** Arterial blood gas analysis.

Parameter	Measured values	Reference values
pH	7.39	7.35-7.45
PaCO_2_	46 mmHg	35-45
PaO_2_	69 mmHg	75-100

**Figure 2:**
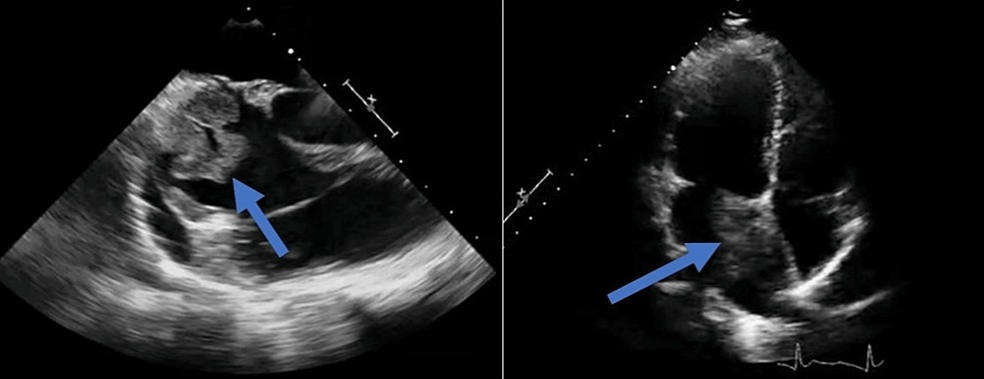
Echocardiogram revealing intracardiac lesion in the right atrium.

**Figure 3:**
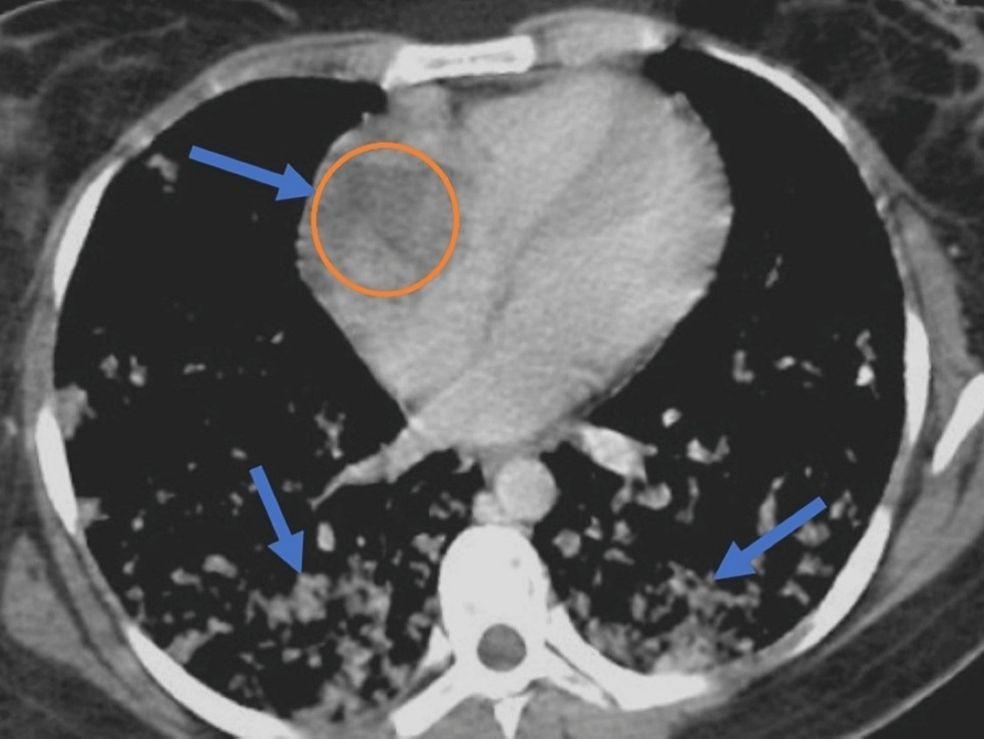
CT chest revealing intracardiac lesion with scattered pulmonary nodules (blue arrows).

**Figure 4:**
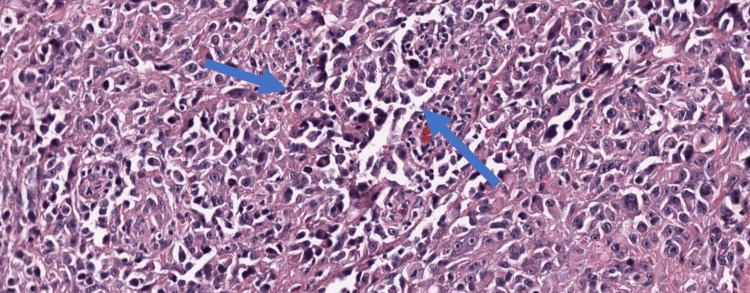
Histopathology of the specimen demonstrating nuclear atypia, abundant mitotic figure, and vascular channels.

Transthoracic echocardiography (TTE) revealed a large mass (5.8cm x 4.2cm) attached to the right atrium, extending into the right ventricle. It appeared to be invading the right atrial wall and protruding into the right ventricular outflow tract (Figure 2). The mass caused mild tricuspid regurgitation and obstruction of the right ventricular outflow tract. Computed tomography (CT) scan of the chest confirmed a large mass originating from the right atrium, invading the right ventricle. Multiple nodules were noted in both lungs, suggesting pulmonary metastases (Figure 3). She was planned for bronchoscopy, and bronchoalveolar lavage sample analysis showed polymorphonuclear cells, macrophage cells, and red blood cells. Biopsy of the lesion indicated an epithelial carcinoma with no necrosis, high mitotic rate, nuclear atypia, and abundant vascular channels suggesting metastatic angiocarcinoma (Figure 4). A positron emission tomography scan showed increased uptake in the cardiac mass, right atrial wall, and multiple lung nodules, indicating active tumor metabolism. Due to the advanced stage of the disease with pulmonary metastasis and involvement of vital cardiac structures, the case was discussed in a multidisciplinary tumor board meeting. The treatment approach was challenging, considering the rarity and aggressive nature of the tumor. A combination of doxorubicin and ifosfamide was initiated as palliative treatment to control tumor growth and metastatic spread. However, the patient showed a limited response, and the disease continued progressing. Given the extensive involvement of the tumor and the risk of perioperative complications, surgical resection was deemed too risky and was not pursued. External beam radiation therapy was considered to palliate symptoms and control local disease, but it was limited due to the proximity of critical cardiac structures. Despite aggressive treatment, the patient's condition deteriorated rapidly, and she succumbed to the disease six months after the initial presentation.

## Discussion

Angiosarcoma is an aggressive malignant endothelial cell tumor with a poor prognosis and accounts for 1%-2% of all soft tissue malignancies [3]. Angiosarcoma can involve any part of the body. Skin, breasts, spleen, bones, and liver are the most affected sites, constituting 60% of the angiosarcoma [4]. However, cardiac and renal involvement is rare. Primary cardiac angiosarcoma constitutes 33% of all primary cardiac neoplasms, with male predominance. The site of the lesion is usually in the right atrium or atrial septum [5]. An analysis of six patients diagnosed with primary cardiac angiosarcoma using surgical or autopsy specimens revealed that all the patients presented with nonspecific signs and symptoms. Dyspnea and chest discomfort were present in all cases, and fatigue and hemoptysis were present in four patients. All cardiac angiosarcoma originated from the right atrium with cardiomegaly in five patients. Pericardial effusion, cardiac mass, and EKG changes were observed in the three patients, and lung METs were noted in five patients. The average survival duration after the presentation was only 180 days. Three patients died because of pulmonary hemorrhage, and pulmonary metastasis was the cause of death in three patients. Four patients were diagnosed with primary cardiac angiosarcoma on autopsy, and two were diagnosed during surgery [6].

Cardiac angiosarcoma is a rare and aggressive tumor with a predilection for early metastasis. Its clinical presentation can be nonspecific, mimicking other cardiac conditions, leading to diagnostic challenges. Echocardiography and CT scans are useful imaging modalities for detecting cardiac masses and evaluating their extent [7]. Biopsy remains essential for confirming the diagnosis. Treating cardiac angiosarcoma is challenging due to its aggressive nature, early metastasis, and involvement of vital cardiac structures [8]. Surgery remains the primary treatment option for localized tumors, but surgical resection is often not feasible in advanced cases with metastasis. Chemotherapy, although not highly effective, is commonly used as a palliative option to control tumor growth and alleviate symptoms. Radiation therapy is another palliative treatment to manage the local disease and reduce tumor burden [9,10].

Despite advances in cancer treatment, the prognosis for cardiac angiosarcoma remains poor, with a median survival of fewer than 12 months from diagnosis. Early detection, prompt multidisciplinary intervention, and innovative treatment strategies are essential for improving patient outcomes [11].

## Conclusions

Cardiac angiosarcoma is a rare and aggressive malignant tumor with a propensity for early metastasis. As clinicians, it is crucial to consider cardiac angiosarcoma in the differential diagnosis of cardiac masses, especially in patients with atypical cardiac symptoms and pulmonary metastasis. Early recognition and a multidisciplinary approach are vital in providing the best possible care and support for patients with this rare and devastating condition. Research efforts are needed to explore novel treatment modalities to enhance patient outcomes.
